# Effect of different heterotrophic plate count methods on the estimation of the composition of the culturable microbial community

**DOI:** 10.7717/peerj.862

**Published:** 2015-03-31

**Authors:** Eva Theres Gensberger, Eva-Maria Gössl, Livio Antonielli, Angela Sessitsch, Tanja Kostić

**Affiliations:** AIT Austrian Institute of Technology GmbH, Bioresources Unit, Tulln, Austria

**Keywords:** Culturable community composition, Temperature, Heterotrophic plate count, Media type, 16S rRNA gene sequence analysis, Microbial water quality assessment

## Abstract

Heterotrophic plate counts (HPC) are routinely determined within the scope of water quality assessment. However, variable HPC methods with different cultivation parameters (i.e., temperature and media type) are applied, which could lead to significant effects in the outcome of the analysis. Therefore the effect of different HPC methods, according to DIN EN ISO 6222 and EPA, on the culturable microbial community composition was investigated by 16S rRNA gene sequence analysis and statistical evaluation was performed. The culturable community composition revealed significant effects assigned to temperature (*p* < 0.01), while for media type no statistical significance was observed. However, the abundance of certain detected bacteria was affected. Lower temperature (22 °C) showed the abundance of naturally occurring Pseudomonadaceae and Aeromonadaceae, whereas at high temperature (37 °C) numerous Enterobacteriaceae, *Citrobacter* spp. and Bacilli were identified. The highest biodiversity was detected at lower temperature, especially on R2A medium. These results indicate that different temperatures (low and high) should be included into HPC measurement and selection of media should, ideally, be adjusted to the monitored water source. Accordingly, it can be inferred that the HPC method is more suitable for continuous monitoring of the same water source than for single assessments of a water sample.

## Introduction

A huge diversity of bacteria can be found in water habitats containing naturally present autochthonous waterborne bacteria but also allochthonous bacteria including opportunistic pathogens derived from fecal contamination of human or animal origin (Pavlov et al., 2004; Cabral, 2010). Therefore, in order to ensure a high quality of water, safe for human consumption, a regular water quality assessment is a prerequisite.

The basis for water quality assessment is outlined in several national and international standards, e.g., in Europe in the Council Directive 98/83/EC on the quality of water intended for human consumption (European Commission, 1998), in the US in the Water Directive (United States Environmental Protection Agency, 2009), in Australia the Drinking Water Guidelines (NHMRC, 2011) and WHO recommendations (WHO, 2011). Even though regulations differ slightly, requirements generally include monitoring of microbial parameters such as fecal indicators (coliforms, *Escherichia coli*, *Enterococcus* spp.), the opportunistic pathogen *Pseudomonas aeruginosa* and determining the heterotrophic plate count (HPC). The HPC is the enumeration of the growth of heterotrophic culturable microorganisms on a non-selective solid medium under defined cultivation conditions. The concept of HPC as a water quality parameter was firstly proposed by Robert Koch in 1883 (Bartram et al., 2003) and to this day is included in most water quality regulations (Allen, Edberg & Reasoner, 2004). The heterotrophic plate count procedure has been subjected to extensive changes to ensure the best possible recovery of heterotrophic organisms (Reasoner, 2004), resulting in variations in methods between countries (Bartram et al., 2003). The commonly used practice for HPC determination is based on the pour-plate method, but also membrane filtration and the spread plate method (Sartory, Gu & Chen, 2008) have been proposed, and results vary across the culture methods. Variability further arises from differences in the resources and temperature of cultivation. For example, DIN EN ISO 6222 (valid Europe-wide e.g., in Austria, Germany and Sweden) prescribes utilization of yeast extract agar (YEA) and incubation at 37 °C and 22 °C for 48 h and 72 h, respectively. The US regulatory framework is more permissive and recommends a range of incubation temperatures (between 20 °C and 40 °C), incubation times (between 48 h and 7 days), and variable formulations of media (e.g., low and high nutrient media) (Reasoner, 2004; Bartram et al., 2004). Many questions that arose at the time when HPC methods were established (including media suitability, relationship between the bacteria in water samples and the corresponding HPC counts) remain the focus of discussion today (Bartram et al., 2003). Some studies experimentally evaluated the effect of varying the cultivation parameters (i.e., media type and temperature) and found differences in plate counts (Lillis & Bissonnette, 2001; Allen, Edberg & Reasoner, 2004; Bartram et al., 2004; Reasoner, 2004; Inomata, Chiba & Hosaka, 2009).

To our best knowledge only limited information is available on the composition of the HPC community obtained from different cultivation media and incubation temperatures. One study addressed the impact of three culture media (YEA, nutrient agar, and R2A) at 20 °C on the cultivable bacterial community (Wernicke, Kampfer & Dott, 1990; Reasoner, 2004). Even though HPC populations were characterized only phenotypically, significant differences between culture media were observed (Reasoner, 2004). Farnleitner et al. (2004) examined diversity profiles of HPC communities by denaturing gradient gel electrophoresis (DGGE) and clearly showed cultivation-dependent variability of culturable HPC community, but the observed DGGE bands were not classified to taxa. As already stated by Burtscher et al. (2009), deeper insights into the community structure as based on HPC will require gene sequencing, because this will lead to better understanding of the method itself and effects of variable cultivation parameters on the microbial characterization of a water sample. We tested two temperatures (22 °C and 37 °C) and two culture media on the composition of the bacterial communities growing on the HPC plates. Two different media: high-nutrient yeast extract agar (YEA), commonly used in the EU, and R2A agar (designated as low-nutrient medium by Reasoner & Geldreich (1985)), recommended in the US (Allen, Edberg & Reasoner, 2004; Reasoner, 2004). The composition of bacteria cultured under these conditions was determined by 16S rRNA gene sequence analysis.

## Materials and Methods

### Water sampling

Water was collected from three private wells from Nappersdorf (N170 and N167) and Tulln an der Donau (IFA) in Lower Austria. A total volume of 5 L was sampled according to DIN EN ISO 19458 in polypropylene plastic bottles (VWR, Vienna, Austria). Samples were transported and stored at 4 °C until further processing (max. 18 h). The three well water samplings were considered as replicates.

### Sample preparation

The membrane filtration method was used for concentration of microorganisms, by filtering a total volume of 1 L through a 0.45 µm nitrocellulose membrane (Millipore, Germany) for each water sample and each tested cultivation condition (Reasoner & Geldreich, 1985). Membrane filters were incubated on yeast extract agar (Sigma Aldrich, Germany) and on R2A (Sigma Aldrich, Seelze, Germany) at 22 °C (72 h incubation) and 37 °C (48 h incubation). The four combinations of cultivation conditions were abbreviated as YEA37, R2A37, YEA22 and R2A22. After incubation on the membrane filter, bacteria were resuspended with 1 ml 0.01% Tween solution, pelleted by centrifugation, and DNA was isolated using the GenElute genomic DNA Kit (Sigma Aldrich, Seelze, Germany), according to the manufacturer’s instructions, and eluted in 100 µl sterile water. The DNA concentration was measured with a Nanodrop spectrophotometer (Thermo Scientific, Scoresby, Victoria, Austria) and confirmed with agarose (1%) gel electrophoresis.

### 16S rRNA amplification and cloning

The 16S rRNA gene was amplified with universal bacterial 16S rRNA oligonucleotide primers 8f (5′–AGAGTTTGATCCTGGCTGAG-3′) and 1520r (5′-AAGGAGGTGATCCAGCCGCA-3′) (Edwards et al., 1989; Massol-Deya et al., 1995). A 25 µl reaction was prepared containing 1x Taq^®^ buffer, 2.5 mM MgCl_2_, 0.2 mM dNTP mix, 0.15 µM of each primer, 1 U of Taq^®^ polymerase (Invitrogen, Lofer, Austria), and 2 µl of template DNA. The cycling conditions were as follows: initial denaturation at 95 °C for 5 min, 25 cycles of 95 °C for 30 s, 54 °C for 1 min, 72 °C for 1 min, and a final elongation at 72 °C for 10 min. PCR products were purified using Sephadex (Sigma Aldrich, Seelze, Germany). Amplicons from three separate PCR amplifications were pooled for subsequent cloning and sequencing analysis. 16S rRNA gene clone libraries were constructed using the StrataClone PCR cloning kit following the manual (Agilent Technologies, Vienna, Austria). White colonies containing the insert were picked from Luria Bertani (LB; Sigma Aldrich, Seelze, Germany) plates containing 100 µg/ml ampicillin (Sigma Aldrich, Seelze, Germany) and 80 µg/ml 5-Bromo-4-chloro-3-indolyl *β*-D-galactopyranoside (X-Gal; Biochem, Lohne, Germany) and then grown overnight at 37 °C in liquid freezing media (Wittenberg et al., 2005) containing ampicillin (12.5 µg/ml). Then an aliquot of 1 µl was used for amplification of the insert using oligonucleotide primers M13f (5′-GTAAAACGACGGCCAG-3′) and M13r (5′-CAGGAAACAGCTATGAC-3′). The reaction mix (50 µl) contained 3 mM MgCl_2_ and 2 U of Taq^®^ polymerase and the same concentration of reaction buffer, dNTP mix, and primers as for the 16S rRNA gene PCR. Cycling conditions included an initial denaturation step at 95 °C for 5 min, 30 cycles of 95 °C for 45 s, 58 °C for 1 min, 72 °C for 2 min, and a final elongation at 72 °C for 10 min.

### Partial 16S rRNA gene sequencing and sequence analysis

M13 PCR products were sent for sequencing to LGC Genomics (Germany) using the standard sequencing primer T3 (5′-AATTAACCCTCACTAAAGGG-3′). Electropherograms were then imported in the Geneious software for peak quality check. Final sequences were generated by manual trimming (Kearse et al., 2012). Sequence data are deposited at Genbank under accession numbers KP706779—KP706794.

Preprocessed sequences and ancillary metadata were analysed using Quantitative Insights Into Microbial Ecology (QIIME) pipeline (Caporaso et al., 2010). Quality filtering consisted of excluding homopolymer runs (>6 nt) and ambiguous bases (>6 nt). Chimera removal and OTU selection were accomplished with USEARCH with a criterion of 0.97 (Edgar, 2010; Edgar et al., 2011). Taxonomy assignment was performed employing the naïve Bayesian RDP classifier with a minimum confidence of 0.8 (Wang et al., 2007) against the latest version (May 15, 2013) of the Greengenes database (http://greengenes.secondgenome.com/). The Greengenes tree was then used for phylogeny-based beta diversity calculations (McDonald et al., 2012)

### Statistical analysis

All data were tested for normality (Shapiro–Wilk) and a log transformation was applied to meet the criteria for normal distribution, as suggested in the publication of Anderson, Ellingsen & McArdle (2006) and implemented in the vegan R package as decostand function.

When data did not pass the normality test again, then nonparametric tests based on permutations were applied for further analysis.

Alpha-diversity metrics based on richness (Chao’s richness estimator) (Chao, 1984) and diversity (Simpson’s diversity index) (Simpson, 1949) were calculated after samples were randomly rarefied to the number of sequences in the poorest sample (Fig. S1).

Good’s coverage estimator was used for estimating the sampling completeness and calculating the probability that a randomly selected amplicon sequence from a sample has already been sequenced (Good, 1953). A permutational *t*-test was used for comparing the alpha diversity of samples (9999 permutations) (Hervé, 2014).

Multivariate analysis of community structure and diversity were performed using: (1) unconstrained ordination offered by Principal Coordinate Analysis (PCoA), (2) constrained multidimensional scaling using Canonical Correspondence Analysis (CCA), (3) permutation test for assessing the significance of the constraints and permutational multivariate analysis of variance (PERMANOVA), (4) indicator value analysis of taxa associated with the grouping factors used as constraints.

The differences between bacterial communities were investigated using the unweighted Unifrac dissimilarity matrix (Lozupone & Knight, 2005) and the ordination methods applied to the matrix calculated in this way. The Unifrac distance was calculated jackknifing read abundance data at the deepest level possible (35 sequences) after 100 reiterations. The PCoA ordination analysis (Gower & Blasius, 2005) was computed in QIIME and plotted using KiNG (Chen, Davis & Richardson, 2009)(point 1), whereas the CCA was calculated and plotted using the vegan R package (point 2). The significance of the grouping factors used as constraints in the CCA was assessed via the permutation test (Oksanen et al., 2013) in the vegan R package. The null hypothesis of no differences between a priori defined groups (i.e., assuming no constraints, as for the PCoA) was investigated using the PERMANOVA approach (Anderson, 2001), implemented in vegan as the ADONIS function and applied to the Unifrac dissimilarity distance matrix (point 3). Taxon-group association analysis was calculated using the indicspecies R package (Cáceres & Legendre, 2009) to determine whether the presence of one OTU was associated either to the R2A or to the YEA medium or to the temperature incubation of water sample analysed. For determining if the OTU relative abundance was different between the samples grouped by medium and temperature, an ANOVA with the FDR method was calculated in QIIME (point 4).

## Results and Discussion

### Bacterial alpha diversity based on 16S rRNA gene sequence analysis

Bacterial diversity and the community composition were evaluated from 563 chimera-checked sequences. This corresponds to an average of 46.9 ± 10.8 (*n* = 12) sequences per cultivation condition and sample, with an average read length of 484.8 bp and a min and max of 268 and 570 bp, respectively. Sequence clustering yielded a total number of 16 (6 ± 2.5) OTUs and the reference sequence of each OTU was used for taxonomic assignment. When grouped by the medium, the number of sequences in R2A was 252 and 311 in YEA (281.5 ± 29.5), corresponding to 15 and 14 OTUs, respectively. Grouped by temperature, HPC plates incubated at 22 °C and 37 °C yielded 269 and 294 (281.5 ± 12.5) sequences, corresponding to 16 and 9 OTUs (12.5 ± 3.5), respectively.

Alpha diversity was measured for different cultivation conditions (i.e., temperature and medium) for observed OTU abundance, Chao1 richness estimator, and Simpson diversity, as well as Good’s coverage estimator (Table 1). The coverage was between 94–97% for the tested cultivation parameters, which occasionally could be considered close to saturation. However, it should be considered, that in general there is a low likelihood for determining low frequency bacteria. This is depicted that the Simpson diversity values were rather low with 57% for the cultivation temperature of 37 °C, representing a low diversity in the microbial community. In comparison alpha diversity measures at 22 °C showed the highest number of OTU’s, Simpson diversity index and the highest richness value. This was confirmed by permutational *t*-test comparison, which resulted in significant effect for temperature categories, 22 °C and 37 °C, for different OTUs (*t* = 2.7116, *p* < 0.05) and between the Simpson’s diversity values (*t* = 3.5, *p* < 0.01). In the study of Wernicke, Kampfer & Dott (1990) the significance of diversity measures (Shannon and Weaver index) were allocated to different tested media, with higher values for R2A than YEA. However, different temperatures were not considered; only 20 °C incubation temperature was tested.

**Table 1:** Alpha diversity values grouped by the medium and temperature. The observed prokaryotic richness and diversity estimates were based on identified OTU clusters from three analyzed water samples (IFA, N167, N170) as replicates.

**Medium**	Observed OTUs	Chao richness estimator	Simpson diversity index %	Coverage %
**R2A**	6.2 ± 3.2	6.7 ± 3.2	66 ± 0.1	95 ± 0.03
**YEA**	5.2 ± 2.2	7.8 ± 8.0	64 ± 0.1	96 ± 0.1
**Temperature (°C)**	Observed OTUs^*^	Chao richness estimator	Simpson diversity index*	Coverage %
**22**	7.3 ± 2.9	10.0 ± 7.5	72 ± 0.1	94 ± 0.1
**37**	4.0 ± 0.9	4.5 ± 1.2	57 ± 0.05	97 ± 0.03

**Notes.**

* Statistically significant differences (*p* < 0.05) were reported according to a nonparametric permutation test using 9999 permutations and the FDR method was used for correcting the comparisons. The *p*-values were significant for the observed OTUs and the Simpson diversity index, for the temperature only.

### Culturable microbial community composition from 16S rRNA gene sequence analysis

The culturable microbial community composition and identified taxa were obtained from 16S rRNA gene sequencing and in total, 16 OTUs (i.e., sequence clusters) were determined. After the taxonomic assignment, the 16 OTUs were summarized at the deepest taxonomic level possible, resulting in 12 taxa within the Firmicutes and the Proteobacteria. Most clones in our study could be affiliated to the family or genus level. One exception was Tax4 (Bacilli), which was resolved only to the class level. The relatively low number of OTUs is likely due to the low resolution of partial 16S rRNA gene sequence, yielding assignments from class to genus level only. Furthermore, the observed composition is restricted to culturable organisms, only representing a small proportion of the total microbial community. This was also observed by Farnleitner et al. (2004), where a maximum of 12 OTUs from culturable HPC bacteria were determined with DGGE. Consequently, it should be taken into account that cultivation-dependent methods such as HPC, might fail to detect rare taxa. Identification of the rare biosphere will require cultivation-independent approaches (Galand et al., 2009). For example, Liu et al. (2012) detected more than 400 OTUs from faucet water by pyrosequencing.

### Effect of cultivation conditions (media type and temperature) on the cultivable microbial composition

**Figure 1:**
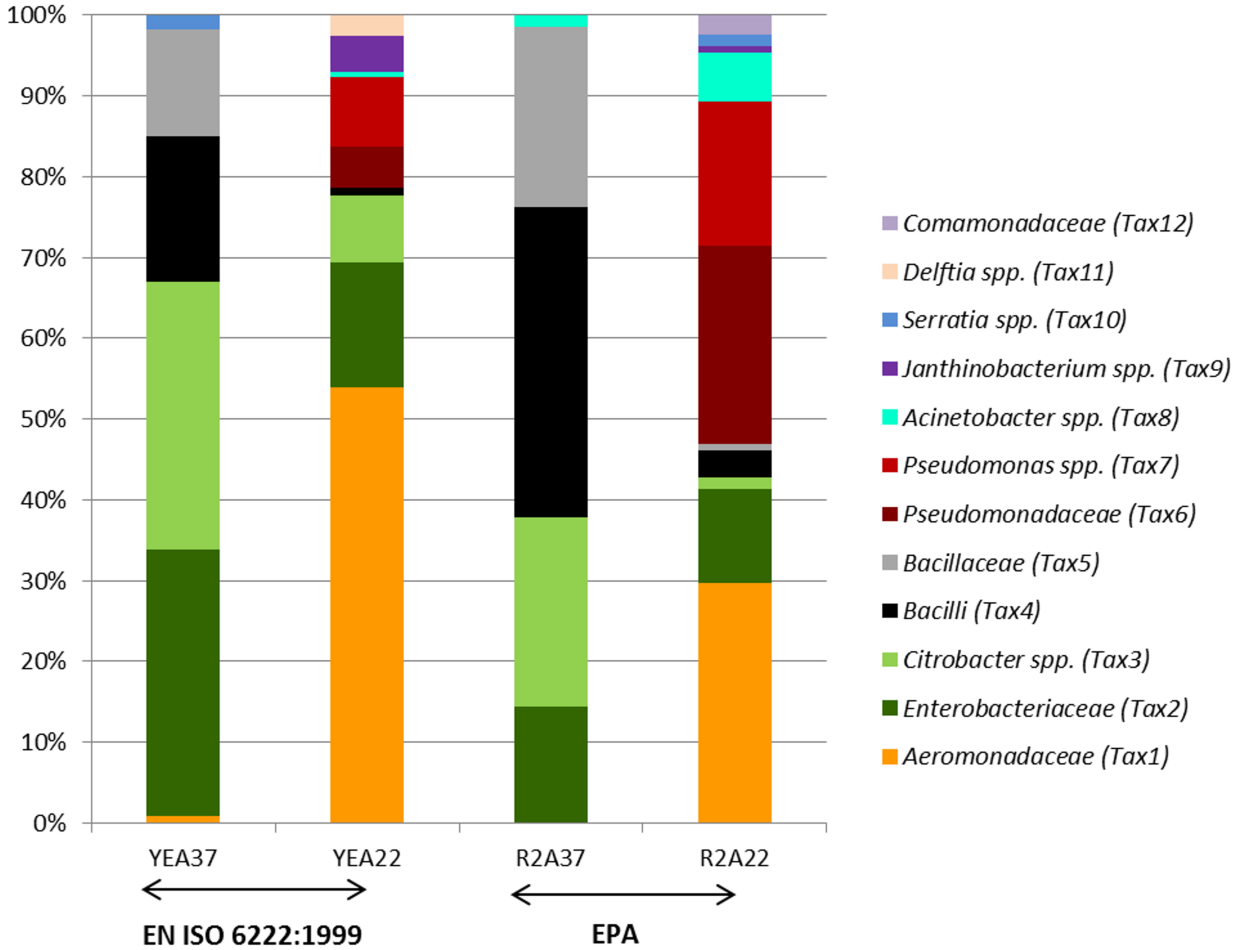
Identification of the community composition from partial sequences of the 16S rRNA gene. Two different HPC methods (EN ISO 6222:1999 and EPA recommendations) and the corresponding cultivation conditions such as medium type (R2A or YEA) and incubation temperature (22 °C and 37 °C) were tested. The community composition for each cultivation treatment is averaged over three water samples. Identified sequence types were assigned to the deepest taxon level possible.

The four cultivation conditions yielded different community compositions, as seen in Fig. 1, according to the prescribed HPC methods such as DIN EN ISO 6222 (YEA and two temperatures) and EPA (R2A and two temperatures). Taxa varied in abundance across cultivation conditions and in some cases taxa that were present in one condition were not detected in another. Thus applying only one cultivation condition (one medium type at one temperature) would miss a considerable part. For example, YEA37 or R2A37 would completely fail to detect Pseudomonadaceae family members (Tax6 and 7), which were abundantly determined from R2A22. The cultivation condition of R2A22 further showed the greatest diversity of taxa and was the only one to yield growth of Comamonadaceae (Tax12). It has been previously reported, that incubation on R2A at lower temperature allows the cultivation of rare, slow-growing bacteria and therefore yielded higher levels of diversity (Reasoner & Geldreich, 1985; Wernicke, Kampfer & Dott, 1990; Reasoner, 2004). R2A was developed by Reasoner & Geldreich (1985) and was designated as low nutrient media since it contains lower carbon concentration and ionic strength (Reasoner & Geldreich, 1985; Allen, Edberg & Reasoner, 2004). Still, R2A nutrient concentrations are significantly higher (800×) than those normally found in water habitats (Hammes et al., 2008), but closer approximates environmental conditions (Allen, Edberg & Reasoner, 2004). In comparison high nutrient media (e.g., YEA) and high temperature was prescribed for the recovery of fecal derived bacteria (Sartory, 2004; Reasoner, 2004).

In our study, the media type seemed to play only a minor role on the recovered community. Almost the same taxa were detected on R2A and YEA, however with different abundances. For example *Acinetobacter* spp. (Tax8) was rarely identified, but resulted in higher numbers on R2A. In comparison Aeromonadaceae (Tax1) seemed to be more frequent on YEA, whereas Pseudomonadaceae (Tax6 and 7) was more abundant on R2A. Also Wernicke, Kampfer & Dott (1990) found that several *Pseudomonas* isolates were more likely to be found on R2A. However, statistical tests (significance tests, PERMANOVA and PcoA) demonstrated no significant effect ascertained to the medium.

In contrast, temperature had an important effect on the prevalence of certain taxonomic groups. Enterobacteriaceae (Tax2), *Citrobacter* spp. (Tax3) and Bacilli (Tax4 and 5) were more abundant at 37 °C, while Pseudomonadaceae (Tax6 and 7) and Aeromonadaceae (Tax1) were more frequent at 22 °C. This was also confirmed by the OTU category significance test, which showed significant associations with 22 °C for the OTU’s assigned to the Aeromonadaceae family (Tax1) (*p* < 0.01) and to Pseudomonadaceae (Tax6 and Tax7) (*p* < 0.05). The only taxon significantly correlated with 37 °C was Tax3 (*Citrobacter* spp.) (*p* < 0.05), belonging to Enterobacteriaceae. For Bacilli (Tax 4 and 5) the mean count was higher at 37 °C (*p*-value <0.01) using the FDR-corrected ANOVA. The abundance of Enterobacteriaceae at 37 °C is in agreement with the earlier hypothesis that body temperature would recover fecally derived organisms, including also some potentially pathogenic members (Allen, Edberg & Reasoner, 2004). Therefore this temperature was defined for HPC analysis. Notably high numbers of *Aeromonas* spp., which are generally not easily detected from water samples by HPC methods (APHA, 1998; Allen, Edberg & Reasoner, 2004), could be ascertained at 22 °C, suggesting that this temperature is a promising candidate for cultivation of this genus from a water sample. Yet more comprehensive studies have to be performed to give further evidence.

**Figure 2:**
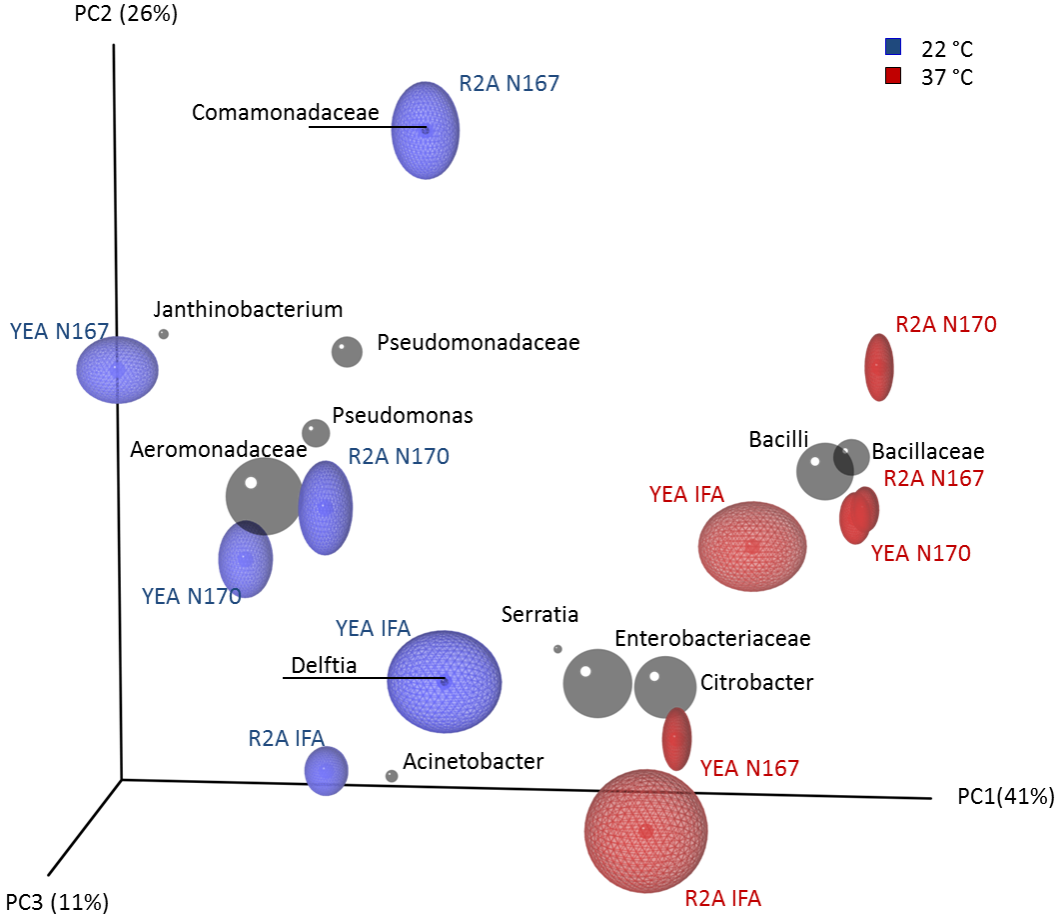
Multivariate analysis by ordination method. The evaluation of statistical significances of the cultivation parameters (i.e., temperature and media type) was performed by Principal Coordinates Analysis (PCoA). The biplots for each taxon (grey spheres) were plotted with diameters proportional to the numbers of assigned sequences. Each sample data point was drawn as a central point, surrounded by a semi-transparent cloud representing the variation in jackknifed Unifrac results. The PCoA plot illustrates clustering of communities according to the temperature of cultivation. Media type (R2A and YEA) showed only differences in the abundances of taxa (cluster sphere).(Aeromonadaceae (Tax1); Enterobacteriaceae (Tax2); *Citrobacter* spp. (Tax3); Bacilli (Tax4); Bacillaceae (Tax5); Pseudomonadaceae (Tax6); *Pseudomonas* spp. (Tax7); *Acinetobacter* spp. (Tax8); *Janthinobacterium* spp. (Tax9); *Serrati*a spp. (Tax10); *Delftia* spp. (Tax11); Comamonadaceae (Tax12))

The effect of temperature was further confirmed with an unsupervised analysis offered by the PCoA ordination method (Fig. 2). The PCoA plot showed clearly the clustering of HPC composition (i.e., sharing of the same taxa) according to the temperature category. The taxa were clearly assigned either to 22 °C or 37 °C. As seen for previous findings by the community composition, for example Pseudomonadaceae (Tax6 and 7) was present at 22 °C and Bacilli (Tax4 and 5) was allocated to 37 °C. Furthermore, taxa such as Comamonadaceae (Tax12) and *Delftia* spp. (Tax11) or *Janthinobacterium* spp. (Tax9) were found exclusively at 22°. In contrast, *Serratia* spp. (Tax10) and *Acinetobacter* spp. (Tax8) could be found at both temperatures (also shown in Fig. 1).

Statistical verification of the findings of PCoA was accomplished by means of PERMANOVA and provided a significant effect for the cultivation temperature (*p* < 0.01, df = 1, pseudo-F = 6.4471), but there was no statistical significance for the media type. A permutation test was also conducted on CCA (Fig. S2) in order to evaluate the significance of constraints factors used, and also in this case a significant effect (*p* < 0.01, df = 1, pseudo-F = 4.1544) was determined for temperature only.

The culturable HPC community composition indicated a certain dependence on temperature and assessing two temperatures (22 °C and 37 °C) together gives a more diverse and complete community composition.

## Conclusions

Our study demonstrated that the choice of cultivation condition can significantly affect the composition and abundance of detected heterotrophic bacteria, which will, in turn, have an influence on the overall outcome of the HPC application for water quality assessment. The most significant effect was observed for temperature, confirming the basic concept that application of two incubation temperatures is of utmost importance. In general, incubation at 22 °C allowed for the detection of more OTUs; in combination with R2A medium, most comprehensive insight into diversity of recovered heterotrophic bacteria is obtained. Media type showed no significant effect in statistical analysis, but results indicated that media may influence the abundances of recovered taxa. Taking into consideration the quantitative nature of HPC analysis, this effect should not be disregarded. The community and native microflora, considering fast-growing and slow-growing organisms in an examined water sample, determine the suitability of the cultivation conditions and wherever practicable should ideally be tested.

Ongoing developments in the field of culturomics raised the proportion of culturable microbial community beyond the conventionally reported 1% (Amann, Ludwig & Schleifer, 1995). However, as demonstrated in the study by Lagier et al. (2012), comprehensive determination of microbial communities with cultivation-based methods remains extremely complex (70 cultivation conditions were needed for the 100% recovery of species from gut microbiome) and beyond the possibilities of routine analysis.

Concluding, the HPC method is more suitable for continuous monitoring of the same water source (i.e., water suppliers), because in this case quality changes, i.e., fluctuations in the counts are measured rather than by determining absolute values. The same HPC method should be used in order to achieve comparable results. However, the single-sporicidal assessment of a water sample might lead to divergent HPC results and biased conclusions according to which HPC method is selected.

## Supplemental Information

10.7717/peerj.862/supp-1 Figure S1 Rarefaction curves Rarefaction analysis for each tested cultivation condition (YEA37, YEA37, R2A37 and R2A22) and water samplings (IFA, N167 and N170) was based on observed OTUs from 16S rRNA sequences at 97% similarity. Samples were plotted in different line styles and blue and red colours assigned to 22 °C and 37 °C temperature condition, respectively. The number of sequences in the less abundant sample was used as high depth value for the calculation.

10.7717/peerj.862/supp-2 Figure S2 Hypothesis driven assumption of constrained model (CCA plot) A permutation test was conducted on CCA for the evaluation of the significance of the constraints factors used (i.e., cultivation parameters (YEA, R2A and the two temperatures) and water sampling replicates (IFA, N167, N170)). Data represented the significance on temperature category (*p* < 0.001, 9999 reiterations). Clusters were primarily formed according to the temperature 22 °C (blue) and 37 °C (red) according to their affiliated taxa. (Tax1—*Aeromonadaceae*; Tax2—*Enterobacteriaceae*; Tax3—*Citrobacter* spp.; Tax4—*Bacilli*; Tax5—*Bacillaceae*; Tax6—*Pseudomonadaceae*; Tax7—*Pseudomonas* spp.; Tax8—*Acinetobacter* spp.; Tax9—*Janthinobacterium* spp.; Tax10—*Serratia* spp.; Tax11—*Delftia* spp.; Tax12—*Comamonadaceae*).

## Additional Information and Declarations

### DNA Deposition

The following information was supplied regarding the deposition of DNA sequences: Sequence data are available from Genbank KP706779–KP706794
