# Supplementary figures and images for: Effect of different heterotrophic plate count methods on the estimation of the composition of the culturable microbial community
Source: PeerJ. 2015 Mar 31;3:e862. doi: 10.7717/peerj.862 (PMC4389272; doi:10.7717/peerj.862)

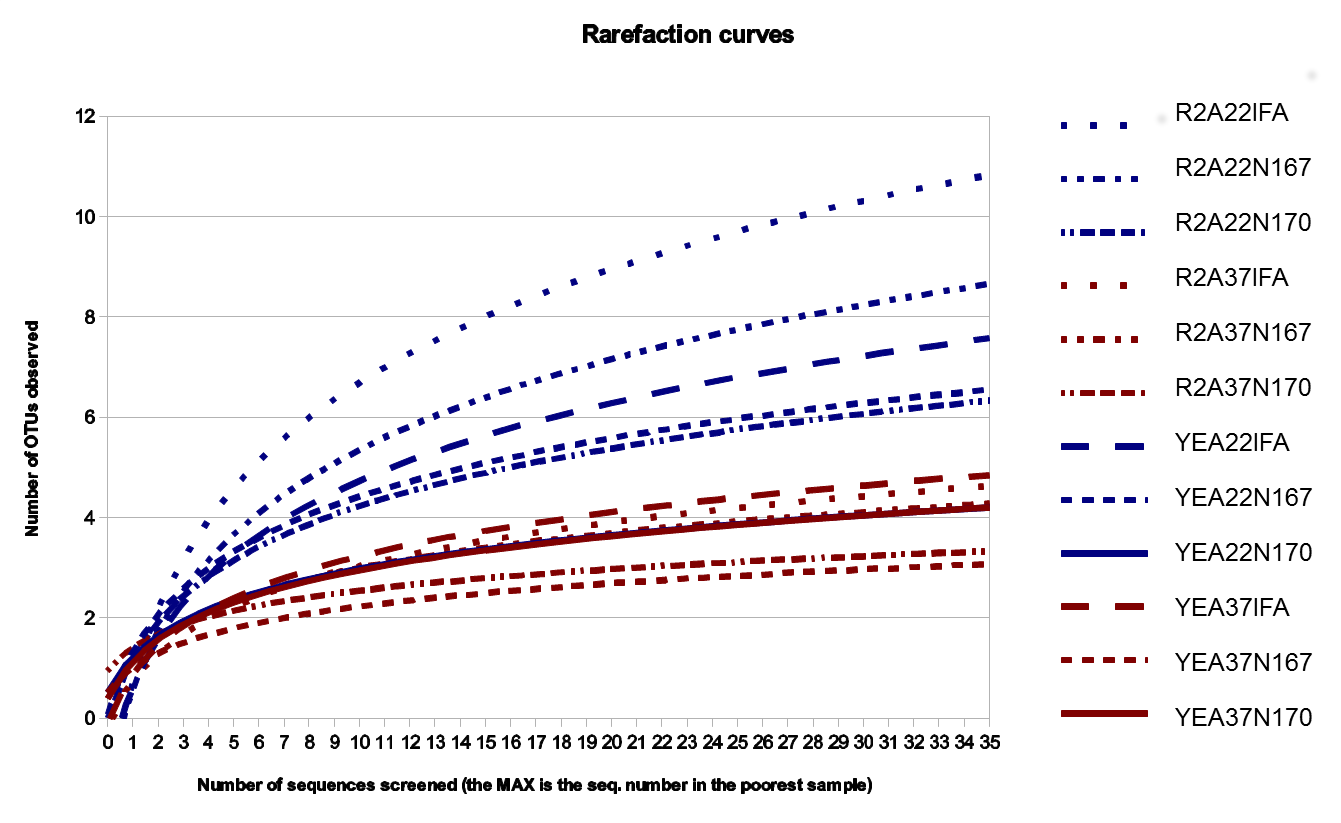

Supplement: Figure S1 — Rarefaction analysis for each tested cultivation condition (YEA37, YEA37, R2A37 and R2A22) and water samplings (IFA, N167 and N170) was based on observed OTUs from 16S rRNA sequences at 97% similarity. Samples were plotted in different line styles and blue and red colours assigned to 22 °C and 37 °C temperature condition, respectively. The number of sequences in the less abundant sample was used as high depth value for the calculation. [file peerj-03-862-s001.png]

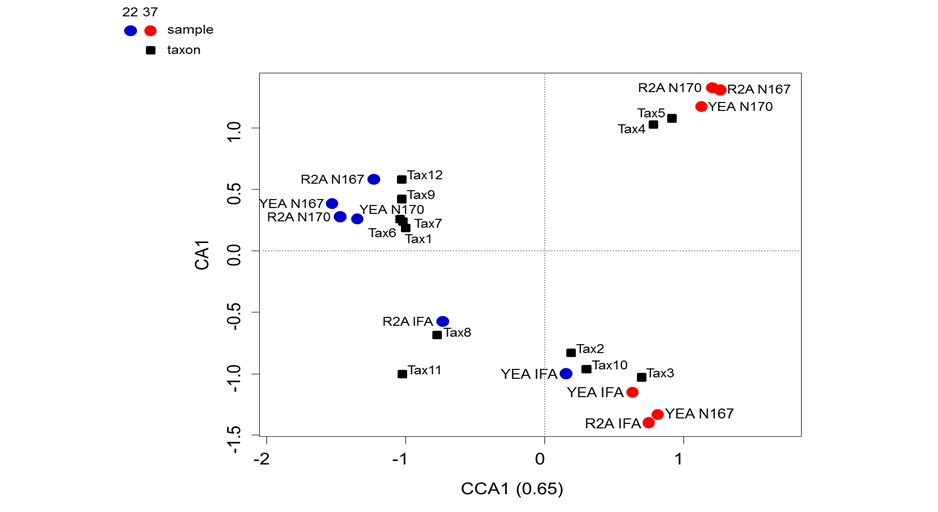

Supplement: Figure S2 — A permutation test was conducted on CCA for the evaluation of the significance of the constraints factors used (i.e., cultivation parameters (YEA, R2A and the two temperatures) and water sampling replicates (IFA, N167, N170)). Data represented the significance on temperature category (p < 0.001, 9999 reiterations). Clusters were primarily formed according to the temperature 22 °C (blue) and 37 °C (red) according to their affiliated taxa. (Tax1—Aeromonadaceae; Tax2—Enterobacteriaceae; Tax3—Citrobacter spp.; Tax4—Bacilli; Tax5—Bacillaceae; Tax6—Pseudomonadaceae; Tax7—Pseudomonas spp.; Tax8—Acinetobacter spp.; Tax9—Janthinobacterium spp.; Tax10—Serratia spp.; Tax11—Delftia spp.; Tax12—Comamonadaceae). [file peerj-03-862-s002.png]
